# Testing the Goodness of Supplementary Feeding to Enhance Population Viability in an Endangered Vulture

**DOI:** 10.1371/journal.pone.0004084

**Published:** 2008-12-30

**Authors:** Daniel Oro, Antoni Margalida, Martina Carrete, Rafael Heredia, José Antonio Donázar

**Affiliations:** 1 IMEDEA (CSIC-UIB), Esporles, Mallorca, Spain; 2 Bearded Vulture Study and Protection Group, El Pont de Suert, Lleida, Spain; 3 Department of Conservation Biology, Estación Biológica de Doñana (CSIC), Sevilla, Spain; 4 Camino del Túnel, Gijón, Spain; University of Zurich, Switzerland

## Abstract

**Background:**

Human-predator conflicts are directly or indirectly threatening many species with extinction. Thus, biologists are urged to find simple solutions to complex situations while avoiding unforeseen conservation outcomes. The provision of supplementary food at artificial feeding sites (AFS) is frequently used in the conservation of scavenger bird populations currently suffering from indirect poisoning, although no scientific studies on its effectiveness have been conducted.

**Methodology/Principal Findings:**

We used a long-term data set of 95 individually marked birds from the largest European core of the endangered bearded vulture (*Gypaetus barbatus*) to test the long-term effects of specific AFS for bearded vultures on their survival rates (by CMR models) and population dynamics (by Monte Carlo simulations) in an area where fatalities derived from illegal poisoning and the use of other toxics like veterinary drugs have increased over the last several years. Our data support the positive relationship between the use of AFS and survival. However, contrary to theoretical predictions (e.g. high and more stable adult survival among long-lived species), the use of AFS increased only survival of pre-adults. Moreover, AFS buffered the effects of illegal poisoning on this age-class, while adult survival decreased over years. Our simulations predicted a maximum value of extinction probability over a time horizon of 50 years. Population projections run with survival rates expected in scenarios without poisoning predicted the situation of least conservation concern, while including only AFS can maintain a large floater surplus that may delay population decline but fails to reduce poisoning risk among adults.

**Conclusions/Significance:**

Although AFS are not effective to save bearded vultures from an expected population decline, they delay population extinction and can be a useful tool for prolonging population viability while combating illegal and indirect poisoning. The eradication of different sources of poisoning is of top priority to ensure the long-term viability of this and many other species.

## Introduction

As the number of species threatened with extinction continues to increase, biologists are asked to identify the factors causing species' declines and to develop management recommendations for their recovery. In this regard, ecological theory predicts that, once phylogeny has been controlled for, increase in extinction risk is associated with an increase in body size and a decrease in fecundity [1], two traits characterizing species with slow life-styles [2]. Thus, sources of extinction risks disturbing the balance between fecundity and survival such as human persecution can be particularly hazardous for these taxa [3]–[4], as has been reported worldwide for many large predators (e.g. [4]–[6]).

Although modern laws in many developed countries have managed to persuade people to abandon direct persecution towards predators, the illegal use of poison or some veterinary drugs as treatment of livestock are still resulting in devastating effects on many populations of specialist or facultative scavenger predators (e.g. [5]–[9]). Indeed, several once-abundant species such as most Old-World vultures are currently considered endangered or critically endangered because of the effects of this unintentional mortality (see [6], [10]). The provision of supplementary food at artificial feeding stations or “vulture restaurants” (hereafter AFS), a well established management tool in the conservation of scavenger populations [11], appears to be a potentially useful solution worldwide. AFS has been frequently used to facilitate the recolonization of abandoned areas [11], or to provide safe food sources in areas where carcasses are baited with poisons to control carnivores (e.g. [12]) or livestock has been treated with veterinary drugs [13]. However, their actual positive effects on the demographic parameters of endangered populations are still hypothetical. To the contrary, negative effects of artificial feeding stations on demographic parameters of some species [14]–[15] are a concern, and prompt us to correctly assess their effect on the demography of target populations.

**Figure 1:**
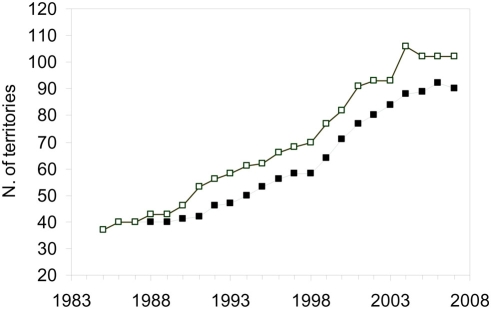
Number of breeding pairs (solid squares) and territories (open squares) of bearded vultures in the Spanish Pyrenees from 1985 to 2007.

The bearded vulture (*Gypaetus barbatus*) is an endangered species in Europe, and the Spanish population, restricted to the Southern part of the Pyrenean Mountains, is its largest stronghold (>80% of the European population). Prohibitions on hunting birds of prey and, more broadly, using poison to control predators may have greatly influenced the recovery of this population, which increased from 40 breeding pairs in the 1980s to ca. 100 breeding pairs in 2007 (Fig. 1). Supplementary feeding through specific AFS (i.e., supplied with food adequate for this species such as lamb legs), the most important management action applied for the conservation of this species, may have also been significant in this recuperation. However, evidence of a direct link between the use of AFS and higher survival rates are merely hypothetical and lack scientific corroboration. Moreover, their actual relevance for population viability through increments in the survival rates of non-territorial birds defies the theoretical low sensitivity of population growth rates to this parameter expected among long-lived species [2]. Yet, the advantage of a predictable food supply in improving population viability remains untested while largely guiding the management of the species and the rest of the scavenging community. Conversely, AFS appear to be discouraging population expansion outside the Pyrenean Mountains and have been related to habitat saturation processes [16], which are triggering negative population effects such as reductions in productivity and changes in mating behaviour [14]–[15].

The Spanish bearded vulture population has been closely monitored since the 1980s, and a relatively large data set on productivity (ca. 30 years, 1,028 breeding records) and individually marked birds (1987–2005, 95 individuals) are available. As we previously mentioned, the population has been managed by opening many AFS. From 1983 to 2002, 5 large (>5,000 kg of lamb legs per year) and 21 small (<3,000 kg of lamb legs per year) AFS were maintained in a 2,100 km^2^ area [14]. These predictable feeding points, where up to 80 birds may congregate during early spring, have fixed non-breeding birds to this area, facilitating an increase in the resighting of marked individuals [17]. Moreover, as in most Iberian regions, the Pyrenean Mountains are suffering increasing levels of illegal poisoning since 1990, which constitute the main cause of mortality for this [18] and other species in the study area. Besides, other indirect sources of poisoning such as antibiotic residues present in carcasses of medicated livestock can be also affecting survival rates of this and other vulture species (G Blanco and J A Lemus pers. comm.; [9]). Thus, this population is a good model for testing demographic consequences of AFS through analysis of survival rates and projections of the population trend. Taking into account the life-history characteristics of the species and based on the general hypothesis of positive effects of predictable food supply on survival, we predict that increments in the illegal use of poison during recent decades [18] have decreased survival rates of bearded vultures. However, this effect would have been buffered by the use of AFS, the use of which varies considerably among individuals although a general pattern is associated with age (young birds are more frequently seen at AFS than older ones [17]). Population projections under different management scenarios in which poisoning has been combated with a variable degree of effectiveness are also presented. In particular, we established the usefulness of AFS as a management tool to mitigate the negative effects of illegal poison and to improve population numbers, discussing potential management alternatives for the well-being of the population.

## Results

### Use of artificial feeding sites and consequences for survival probabilities

**Figure 2:**
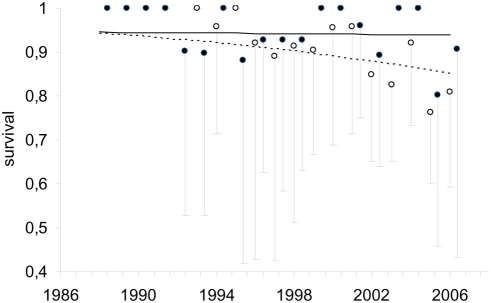
Variation in survival rates of adult (>5y and older; open dots) and young birds (4y and younger; solid dots) with time and age using the parameters obtained with the model [φ_(1_4+5)+T_, *p_A_*]. Mean values and 95% lower confidence intervals are shown, as well as the linear negative trends for adults (dashed line) and young (solid line). Survival rates estimated as 1 were actually estimable parameters, i.e. years in which all individuals survived. Note that the two trends were parallel in the logit scale.

Our best model, accounting for 85% of the weight of all models, included the effects of age, time and the intensity of use of AFS on survival, and of age on resighting rates (model 1, Table 1). Interestingly, the addition of the individual covariate describing the intensity of use of AFS greatly improved the models without this factor (models 7–12, Table 1). However, the use of AFS decreased with bird age (Generalized Linear Mixed Model: age: F_1,386_ *= *56.29, P*<*0.0001), even after controlling for individual variability. Thus, survival was equal for birds of ages up to 4y old (the age classes more frequently seen at AFS) and remained constant over time (0.944±0.012). Conversely, survival of older birds (≥5y old) decreased linearly with time (see Fig. 2), with an average value of 0.878 (±0.014). Other models, including higher number of age classes (results not shown) or different combinations of age and time, behaved worse (Table 1).

**Table 1:** Models of survival and resighting probabilities for bearded vultures in the Spanish Pyrenees.

Model	Survival	Resighting	np	DEV	QAICc	ΔQAICc	WQAICc
**1**	**1_4+5T+**AFS	**A**	**6**	**500.251**	**512.410**	**0.000**	**0.849**
2	1_4+5+AFS	A	5	506.920	517.033	4.623	0.084
3	1_4+5+AFS	1_4+5	5	508.600	518.713	6.303	0.036
4	(1_4+5)T+AFS	A	6	507.877	520.036	7.626	0.019
5	A+AFS	A	5	511.760	521.874	9.463	0.007
6	1_4+5+AFS	5a	8	506.470	522.744	10.334	0.005
7	1_4+5	A	4	553.113	561.113	48.702	0.000
8	1_4+5T	A	5	552.697	562.697	50.287	0.000
9	(1_4+5)T	A	5	552.764	562.764	50.354	0.000
10	1_4+5	1_4+5	4	554.948	562.948	50.537	0.000
11	A	A	4	556.091	564.091	51.680	0.000
12	1_4+5	5a	7	550.799	564.799	52.388	0.000
13	·	A	3	559.284	565.284	52.874	0.000
14	A	1_4+5	4	557.659	565.659	53.248	0.000
15	6a	A	8	550.842	566.842	54.432	0.000
16	·	5a	6	556.559	568.559	56.149	0.000
17	5a	5a	10	549.442	569.442	57.032	0.000
18	5a	A	7	556.741	570.741	58.331	0.000
19	6a	6a	12	548.337	572.337	59.927	0.000
20	[1_4+5]+t	A	22	532.584	576.584	64.174	0.000
21	·	·	2	574.115	578.115	65.705	0.000
22	t	A	21	536.631	578.631	66.221	0.000
23	t	5a	24	533.811	581.811	69.401	0.000
24	5a+t	5a	28	528.235	584.235	71.825	0.000
25	(1_4)T+5	A	5	574.537	584.537	72.127	0.000
26	1_4+5*t	A	18	551.007	588.833	76.423	0.000
27	[1_4+5]*t	A	36	517.177	589.177	76.767	0.000
28	5a*t	5a*t	107	431.466	645.466	133.056	0.000
29	5a*t	A	82	487.020	651.020	138.610	0.000
30	T	5a*t	94	478.640	666.640	154.230	0.000

Time and age were introduced as covariates, either as changes without precise patterns (noted by *t* and *a*, respectively) or changes with a trend (noted by *T* and *A*, respectively). Effects of artificial feeding sites on survival (noted by *AFS*) were introduced as an individual covariate. Models are ranked following QAICc values. np = number of identifiable parameters; DEV = deviance of each model; QAICc = corrected quasi-AIC value for avoiding bias in small samples; ΔQAICc = difference of QAICc value with respect to the best model; WQAICc = weight of each model with respect to the bulk of models tested. “·”, “^*^”and “+” represent mean constancy, interaction and additive effects, respectively. In bold, the final selected model, with ΔAICc<4 relative to the rest of models.

Our results support the hypothesis of improvement of survival rates associated with the use of AFS. Indeed, using the beta parameters of the selected model (model 1 Table 1), survival estimates for the two main age-classes at the beginning of the study period (in 1987 when the effect of poisoning was apparently less marked and AFS were not available) were 0.787 (±0.018) and 0.961 (±0.019) for young (the age-class using AFS) and adults, respectively.

### Population trajectories under different management scenarios

**Figure 3:**
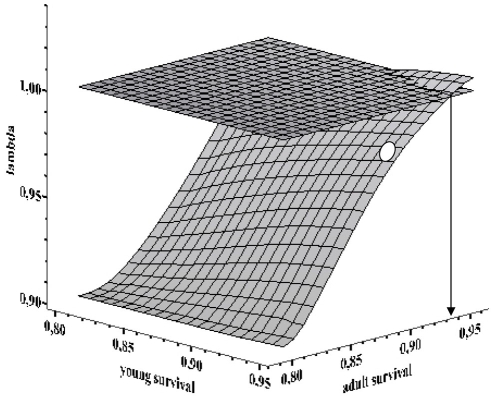
Response of population growth rate (λ) of Spanish bearded vultures to changes in survival of young and adult birds. For each combination of two parameters, a stochastic population simulation was run over 50 years. The horizontal plane shows the population stability, i.e. the area above that plane indicates the combination of survival parameters with positive growth. The open dot shows the actual combination of parameters estimated by capture-recapture modelling, while the vertical arrow shows the minimum pre-adult and adult survival to attain a positive population growth rate.

The current combination of demographic parameters of the Spanish bearded vulture population (scenario 1, Table 2) gave a λ = 0.961 (±0.002). When we explored how this rate changed within a range of different combinations of survival scenarios (all other vital rates remained equal as in further prospective models, see below), we found that the population increased (λ>1) only with very high values of adult survival (>0.93, Fig. 3). To the contrary, λ showed low sensitivity to changes in survival of young (Fig. 3).

**Table 2:** Survival and fertility parameters used in Monte Carlo simulations for estimating extinction risk in prospective models for the bearded vulture in the Spanish Pyrenees depending on the scenario considered.

Parameters	AFS			No AFS	
	Poisoning		No poisoning	Poisoning	No poisoning
	Scenario 1	Scenario 2	Scenario 3	Scenario 4	Scenario 5
Young survival	0.944 (0.012)	0.944 (0.012)	0.944 (0.012)	0.787 (0.018)	0.787 (0.018)
Adult survival	0.878 (0.014)	φ_(t)_·0.264+*rand*·0.241ξ	0.961 (0.019)	0.878 (0.014)	0.961 (0.019)
Fertility§	0.7359e–0.0056·PD*				

Survival was separated for younger birds and a class grouping pre-adult and adult birds. In brackets, SE of the estimates.

ξ Initial value _t = 1_ = 0.961.

* Density-dependence function (PD = population density).

§ Source: Carrete et al. 2006.

**Figure 4:**
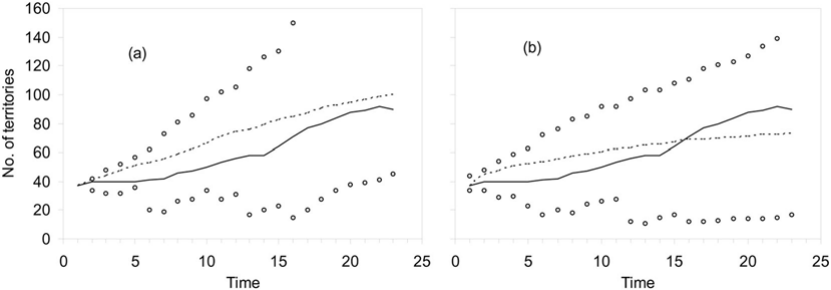
Retrospective analysis of the dynamics of the Spanish population of bearded vultures in the Pyrenees during 1985–2007. Simulations were carried out using estimated values of (a) actual survival rates, i.e. with the effects of poison and AFS; and (b) survival with poison effects but without AFS. Dashed lines show the mean value of stochastic trajectories using Monte Carlo simulations, while open dots are the maximum and minimum values of that run. For comparison, we show the observed number of breeding territories through time (solid lines).

**Figure 5:**
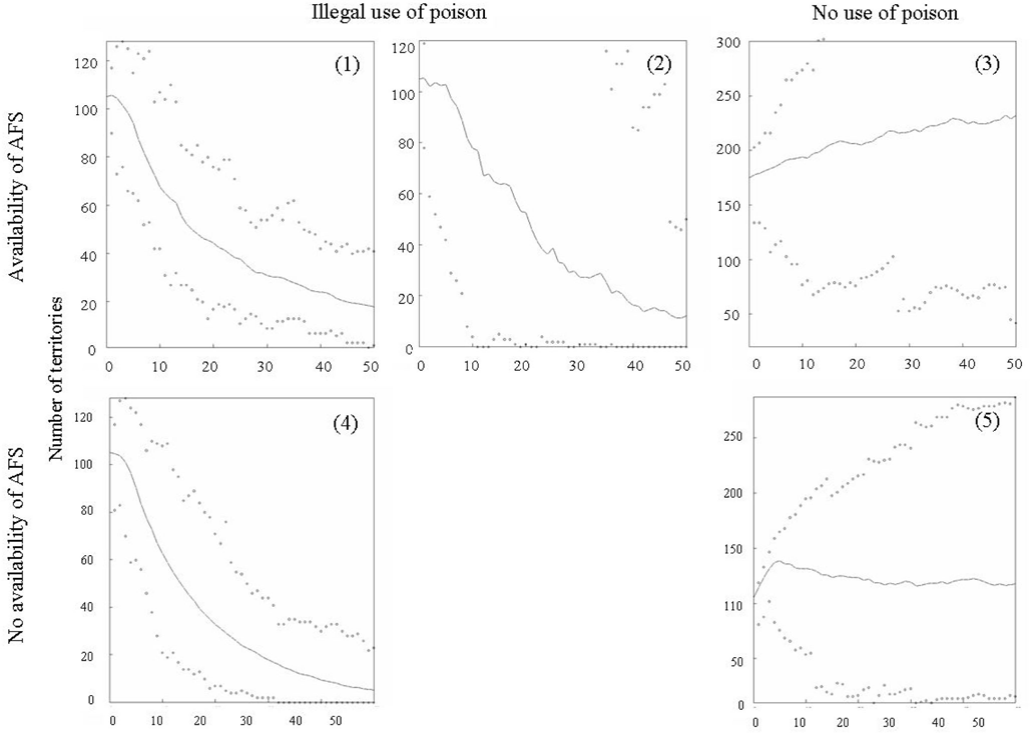
Stochastic population projections estimated through Monte Carlo simulations (50y of temporal window, 500 runs) for some of the scenarios considered: (a) actual values of adult survival without temporal autocorrelation in poisoning, (b) actual values of adult survival with temporal autocorrelation in poisoning, (c) no impact of poisoning, assuming that young and adult survival were equal, and (d) no impact of poisoning, assuming that survival of young and adults were the same t (see text for details). Lines are mean values of the stochastic runs for each time step, while dots shows the maximum and minimum values of those runs. Projections were carried out on density of females of any age, which have different scales in each graph.

Retrospective analysis showed that simulations performed using actual survival estimates (scenario 1 Table 2) fitted quite well with the observed dynamics of the breeding population (mainly at the end of the time series, Fig. 4a), despite the uncertainty of the demographic estimates of the model, and the way they potentially changed with population density (both positively and negatively; author's unpubl. data). Retrospective simulations without the positive effects of AFS on young survival but no effects of poisoning on adult survival rates (scenario 5 Table 2) yielded a lower number of territories than observed in the study population (71 vs. 80 territories, Fig. 4b). However, population trends were quiet similar in all cases (scenario 1: λ = 0.961, scenario 5: λ = 1.000, see Fig. 5, observed: λ = 1.048±0.049) suggesting that population consequences of the progressive reduction in adult survival rates could have been buffered by increments in young survival rates associated with the use of AFS.

#### Availability of AFS

The three scenarios considered within this group of simulations showed different patterns associated with the effects of poisoning on survival (Table 2). When survival of adults decreases because of poisoning and survival of young increases due to their use of AFS (scenarios 1 and 2, Fig. 5), population trajectories decreased over years (scenario 1: λ = 0.961±0.002; scenario 2: λ = 0.932±0.002). However, extinction probabilities were not equal in the first and the second scenarios. When the impact of poisoning was stochastic with no clear trend over time (scenario 1 Table 2), probabilities of extinction were nil, although some trajectories attained the quasi-extinction threshold (scenario 1 Fig. 5). Conversely, ARIMA analysis indicated that the way in which poisoning impacted our bearded vulture population (i.e., the colour of the environmental noise) depended on the age-class considered. For instance, the decrease of survival among birds older than 4y old during 1986–2007 (*a* = 0.264, *b* = 0.241) showed a long-term temporal autocorrelation (i.e., red noise), whereas for birds up to 4y old (*a* = −0.018, *b* = 0.250) survival exhibited a short-term temporal autocorrelation (i.e., blue noise). This temporal autocorrelation in the impact of poisoning, the most realistic situation (scenario 2 Table 2), increased the extinction probabilities of the whole population to its maximum value (*p_e_*(*t* = 50) = 1, mean extinction time: 10.2 years±0.28, see scenario 2 Fig. 5). Note that for these two poison-impacted scenarios the mean trajectories were quite similar, except for the largest variability expected in population trajectories when the impact of poisoning was temporally correlated (scenario 2 Fig. 5).

Increments in the survival rate of adult birds up to that expected when poisoning was near nil (at the beginning of the time series, scenario 3 Table 2) combined with a high survival rate of young (maintained through AFS) predicted a marked increment in the number of breeding territories (λ = 1.014±0.001; scenario 3 Fig. 5).

#### No availability of AFS

In the two final scenarios we considered population trajectories under different pressures of poisoning but no availability of AFS. Populations suffering from negative effects of poisoning on survival rates but not managed with AFS (scenario 4 Table 2) showed a lower population growth rate and a slightly higher probability of extinction in the time horizon of 50y than its most conservative counterpart (scenario 1: λ = 0.961±0.002, *p_e_*(*t* = 50) = 0; scenario 4: λ = 0.932±0.002, *p_e_*(*t* = 50) = 0.05; Fig. 5). However, in a scenario of no poisoning and no AFS (scenario 5 Table 2), population numbers are somewhat lower although still rather stable (scenario 5: λ = 1.000±0.001; scenario 3: λ = 1.016±0.001; Fig. 5).

## Discussion

### Human interference, age-specific survival and population dynamics

Ecological theory predicts that survival of long-lived species should increase gradually with age because 1) individuals improve their competitive skills or change from vagrant life-styles to more sedentary ones after territory acquisition, and/or 2) natural selection progressively eliminates low quality individuals [19]. Even when the underlying mechanisms may be of great interest in understanding the evolution of life histories, it is very difficult to correctly separate one mechanism from another [19]–[20]. All in all, consequences for population dynamics are similar: population growth rates among long-lived species are expected to be highly sensitive to changes in adult survival, so natural selection might have minimized variation in this parameter to ensure population stability [2]. However, our results show how survival of bearded vultures changes through a bird's lifespan in an unnatural way, with non-adult birds (<5y old) having higher and more constant estimates than adults (0.944 and 0.878, respectively, see Fig. 2). Indeed, non-adult birds from this study population show higher survival rates than bearded vultures of the same age-class from the Alps, where there is no availability of AFS [21]. Two main aspects seem to have been directly responsible for this outcome, namely: the opening of AFS (directly tested through individual and age-specific frequencies of visits), and the increment of unintentional poisoning (indirectly tested through a temporal trend in survival). Thus, human activities, both through apparently well-intentioned and malicious actions, can perturb evolutionary forces promoting unexpected changes in survival patterns and, therefore, demographic dynamics.

Our results show that although the frequency at which each bearded vulture was seen at AFS was highly variable there was a trend associated with age (lower use among adults than non-adults). Indeed, both variables (individual frequency of visits to AFS and age) were included in survival models offering the first scientific support to the actual positive effect of AFS on survival rates of a long-lived species. Moreover, the different temporal pattern of survival rates of young (constant) compared with adults (negative) suggests that AFS may be alleviating high mortality risks associated with poisoning [9], [18]. Conversely, most adult birds, which are tied to a breeding territory and have different nutritional requirements during the breeding season [22], must obtain their food by prospecting the landscape and are hence less dependent on AFS and likely more exposed to poisoning. This scenario is probably more obvious after the Bovine Spongiform Encephalopathy crisis, in which the national Spanish law banned farmers to deliver dead cow, sheep and goats into AFS [23].

Population projections under scenarios of poisoning did not forecast a positive outcome for the most important European core of this species. When the impact of poisoning on adult survival was stochastic, probabilities of extinction for the population were nil, although some trajectories achieved the quasi-extinction threshold. Of even greater concern, extinction probabilities for the population increased when the effect of poisoning on adult survival followed a temporal autocorrelation, the most probable scenario. Interestingly, both trajectories were relatively stable in early years, probably due to the buffer capacity of recruits resulting from the very high survival of young birds. Conversely, population projections run with survival of adults not affected by illegal poisoning predicted better situations, with larger population increments in scenarios of maintenance of AFS, when survival rates of young birds are also improved. The counter-scenario, no poisoning and no availability of AFS, predicts slightly lower population sizes but with near stable trajectories. Consequently, an important management action intuitively used to reduce the negative effects of poisoning such as the opening of AFS appears to be not as effective as expected in saving threatened populations from future negative trends. As in many other long-lived species, survival of adults is the key demographic parameter contributing most to the projected population growth rate (e.g. [2]), and management actions should be directed to improve it. However, in the short term, AFS can maintain a large floater surplus that may delay population decline (see above). It is understood that even when supplies to AFS can be quickly changed to antibiotic free carcasses, the eradication of the illegal use of poison is neither easy nor time efficient, and hence measures taken to allow more time for the application of proper and more effective management actions can be a useful instrument for conservation. Thus, efforts to determine adequate tools to reduce poisoning risk among adults are crucial as well. In this sense, experimental work is needed to test the effectiveness of smaller, less predictable AFS located near breeding territories to enhance adult survival while avoiding large aggregations of non-adult birds in their surrounding.

It is worth noting that the reliability of predictive models depends on the robustness of demographic rates and the number of known parameters for each age class. In our case, survival estimates by age classes and fecundity [15] were available and reliable. Nevertheless, some parameters were unknown, mainly the recruitment curve and how it changed with variations in density [24]. Thus, the structure of the model was a compromise between complexity (due to known demographic patterns such as age-dependent survival or density-dependent fecundity), and simplicity (due to the unknown parameters such as percentage of breeders at each age-class) [24]. However, several goals to ensure the maximum reliability at predictive power of extinction risk were achieved in our modelling by incorporating uncertainty in parameter estimates and stochasticity in population dynamics [24], [25]. Finally, it is important to note that demographic consequences of artificial increments in survival are limited to those included in our hypothetical scenarios. Complex aspects linked to alterations in natural selection pressures should also be taken into account [26] since a large proportion of young that in more “natural” situations would have died (low quality individuals [19]–[20]), are now potentially recruited into the breeding population. Moreover, if only AFS-maintained birds are progressively selected, population can become more dependent on human-supplied food than previously thought.

### Usefulness of artificial feeding sites for the conservation of endangered populations

Supplementary feeding to wild birds is a widespread practice that may alter the natural dynamics of food supply, representing a major intervention in avian ecology. Indeed, supplementary feeding has the potential to change long-term population dynamics and distribution ranges of many species (for a revision see [27]). Therefore, policy makers and managers have found in supplementary feeding actions a common, straightforward solution to many different conservation challenges of endangered populations, including increasing breeding success (e.g. [28]), providing safe food in areas were carcasses are poisoned or contaminated with veterinary drugs (e.g. [13]), promoting the recolonization of abandoned areas [11] and aiding in reintroduction programs [29]. However, recent works have shown that supplementary feeding can have negative effects for both target (e.g. [9], [14]–[15], [26], [30]) and non-target populations (for a revision, see [27]), bringing into question their potential benefits for community and population dynamics.

To our knowledge, the Spanish population of bearded vultures represents one of the few cases for which both positive and negative outcomes of artificial feeding sites have being carefully weighed using a population dynamics approach. Recent studies have shown that supplementary feeding can have several negative effects on this population such as territory compression and coexistence between breeders and floaters [14], [16], and changes in the mating system [15] reducing territory quality (because of intraspecific interactions) and population productivity [14]–[15]. Present results, however, support their usefulness as temporal tools to maintain individuals while more complex objectives such as the eradication of illegal poisoning from the field are achieved. However, the maintenance of AFS should not distract managers from prioritizing the long-term viability of this and many other species by eradicating illegal poison use. Taking into account that one of the main threats for this population is its restricted geographic range, AFS can be used as a very specific tool for the recovery of the population in peripheral areas, and to promote the colonization of suitable unoccupied areas outside the Pyrenees. Although beyond the scope of this paper and awaiting scientific support, this latter possibility should be considered a potential positive aspect of AFS on species conservation.

## Materials and Methods

### Study species and population

The bearded vulture is a large, solitary, and territorial scavenger with a diet largely based on bones of domestic and wild ungulates. However, it also scavenges on wild animals such as birds, small carnivores, lagomorphs and small mammals during breeding to feed nestlings, thus facing potential risks of poisoning [18]. After fledging, young bearded vultures disperse from natal territories moving across the Pyrenees and largely aggregating at AFS [17]. In general, the species reaches adult plumage at 5–6 years old (females before males, [31]), and is first-time-paired and become territorial at an average age of 6.5y old. However, mean age of first breeding is 8.1y, and mean age of first successful breeding is 11.4y [32]. Clutch size is usually two eggs, but only one chick survives due to sibling aggression [33].

Illegal poisoning is currently the primary cause of mortality for the species in the Pyrenees. In this sense, samples from bearded vultures found dead or injured were analyzed by the Wildlife Forensic Laboratory (Madrid) for the Ecotoxicology Working Group (Ministry of Environment, Madrid), where post-mortem interval, routine bacteriology and histopathology, and toxicological investigations were performed. Poisoning was recorded when intentional exposure to a toxic substance was confirmed. All cases of strychnine toxicosis and of organophosphate and carbamate pesticides in association with suspected bait in the gastrointestinal tract, regardless of concentration, were considered to be poisonings [18]. Results suggested that 31.4% (n = 16) of bearded vultures found dead between 1955 and 1986 were poisoned while between 1987 and 2006 the proportion of bearded vultures poisoned was 56.3% (n = 38). Of even greater concern, when a subsample of radio-tracked individuals are taken into account, 90% of bearded vultures found dead from 1987 to 2006 (n = 20) were poisoned. Thus, there has been a significant increase in the use of illegal poison during the last several years [18]. Besides, recent studies suggest that, as recorded with other scavenger species [9], bearded vultures could be suffering from ingestion of antibiotic residues present in stabled livestock which are supplied to AFS. This information warns on potential consequences of this unsuspected form of unintentional poisoning on the breeding population of bearded vultures (G Blanco and J A Lemus pers. comm.).

### Field work

#### Population monitoring

The European range of the bearded vultures covers the Pyrenees, Alps, Corsica and Crete. Contrary to the other regions, the Southern face of the Pyrenees has been intensively monitored within the framework of the Species' Recovery Plan in the Autonomous Communities of the Basque Country, Navarra, Aragón and Catalonia. Here, programs to monitor population trends, breeding parameters, and survival rates (including a specific capture-mark-resighting sub-programme) have been performed. Although birds move several kilometres during the dispersal period, the bulk of the non-breeding fraction is largely attached to the AFS just opened within this range [17]. Regrettably, while the Northern face of the Pyrenees (the French population) has also been well monitored through the years, no capture-mark-resighting programme has been performed. Thus, our study focused on the Spanish population alone (no breeding territories scattered outside the Pyrenees have been recorded in Spain). However, as this is the largest population, and where non-breeding birds aggregate, results may be representative of the entire Pyrenean core.

During 1985 to 2007, from early November to August, all known potential territories were visited (2–3 visits/breeding season) to search for signs of occupancy (territorial and/or courtship activity, nest arrangement/building), and to record reproductive parameters. We considered a territory as occupied when a pair was present breeding and/or showing territorial behaviour. We considered a controlled pair or territory as successfully bred when a fledgling was observed during the last part of the breeding period (see [34] for further details).

#### Capture and resighting

During 1987 to 2005, 95 birds of different ages (see below for details) were captured, marked with darvic bands and wing tags and safely released in the study area (for details on capture and marking procedures, see [35]). Each individual was provided with two wing tags with the same alphanumeric code (one per wing) to reduce the probability of being non-identifiable after losing one mark.

From 1988 to 2006, marked individuals were searched for by visiting AFS located within the study area (mainly large AFS) and territories [17]. Only resighting carried out during the first period of each year (from early January to late April) were taken into account to meet the requisite of capture-recapture modelling of having longer non-recapturing seasons than capturing seasons (e.g. [36]–[37]).

### Resighting and survival estimates

#### Loss of wing tags

Since one of the key assumptions of CMR models is that marks are never lost, we explored such potential bias in our study. From the 21 tagged birds found dead during the study period, only two (at 5 and 6y old) had lost one of the two wing marks while the rest (19 individuals) retained the two marks. Age distribution of birds found dead was not biased (median = 6y old, range = 1–16). Using birds seen for the last time (i.e. never seen again, n = 26), 5 still retained one mark at older ages (range = 11–20y old) while the others (21) retained the two marks (range = 8–17y old). Finally, it is worth noting that 6 birds whose wing bands were lost were identified at AFS and breeding territories through their engraved rings by using telescopes. We thus concluded that the potential bias of mark lost in our study should be relatively low.

#### Resighting probabilities

Birds were grouped by their age at release as fledglings (54%) or by their plumage characteristics, into 7 additional age-classes (from 1y old to older than 6y old [31]). Thus, the age of each group was fitted in all models to avoid extra parameters for estimation. Several modelling approaches were possible with the data set available, since some radio-tracked birds were found dead while others were recorded alive. However, the existence of several age-groups at release, the low number of recoveries and the high resighting probabilities (see Results below) advised us to use simple uni-state, live-resighting models, which are also more appropriate for testing some biological hypotheses such as temporal variation in survival.

Basic assumptions of CMR models were assessed through Goodness-of-Fit Test of the Cormack-Jolly-Seber (CJS) model [φ_t_, *p*_t_], using only birds released as fledglings (n = 51 individuals). Although the global test did not show a deviation from the CJS model (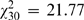, P = 0.862), the TEST2.CT was statistically significant (N(0,1) signed statistic for trap-dependence = −3.1376, p = 0.0017), indicating a trap-happiness phenomenon. Due to the low number of birds released each year, most of the contingency tables had frequencies that were too low and their chi-square tests were not always reliable.

Trap-happiness was likely because some young birds visit the AFS more frequently during their first years of life than others, generating heterogeneity in their resighting probabilities. Moreover, there is a general pattern of reduction in the use of AFS with age, mainly from 6y old and older when the probability of territory acquisition increases [17], [32]. Thus, to test for such age effects on resighting probabilities, we progressively eliminated the resights performed at different ages (from 1y old to 6y old) from the original data set to run GOF tests for each reduced data set. The GOF test for birds of 5y old and older was the only one with a TEST2.CT that was not significant (N(0,1) signed statistic for trap-dependence = −1.8942, P = 0.0582), supporting previous predictions. We thus began with a general model [φ_t_, *p*_5a*t_], with no inflation factor. We then progressively reduced the number of age classes in resighting probabilities for testing for a simpler age-structure in this parameter, ending with a model with no age effects (see Results). All models were fitted using E-Surge software, which allows for the testing of individual covariates [38].

#### Survival probabilities

Age was introduced to test for variations in survival with this individual covariate, with no specific trend or following a trend, as it has been found in other demographic studies (e.g. juveniles showing lower survival than adults, see [21]). Thus, linear and quadratic trends at the logit scale (noted by A and A2 in models, respectively) for variations in age in survival and resighting probabilities were tested. Many models testing potential effects of age (not necessarily showing a trend as in the previous models) grouped the younger age-classes (i.e. birds up to 4y old, noted as 1_4 in models) while the older age-classes were kept separate (i.e. birds of 5y old onwards, noted as 5 in models). Additional groupings also based on ecological knowledge of the species were further tested, namely: birds up to 5y old (noted as 1–5 in models), birds from 6 to 8y old, and individuals being >8y old. Roughly, these categories correspond to immature (non-adult) birds, non-breeding adults and territorial breeding adults (see [32]), respectively. This allowed us to avoid over-parameterized models with high numbers of age-classes, a problem that did not occur when age was introduced as a trend.

After assessing changes in survival associated with age, we tested whether the survival of bearded vultures showed a temporal decrease (i.e., time as a linear trend in the logit scale, noted as T in models) that reflect increments in poisoning during the last two decades (see above). According to our previous prediction of reduced use of AFS as the bird ages, we tested whether this temporal effect of poisoning was stronger for adult (>5y old birds) than for young individuals. Thus, additive effects of age-class (young vs. adult) and time were tested in both survival and resighting probabilities. To test the actual positive effect of AFS on individual survival, we obtained an individual rate of use of AFS corrected by age, survival and resighting probabilities. Thus, we calculated the proportion of times each individual was recorded at AFS out of the total number of censuses performed annually. These values, standardized by subtracting the mean value expected for each age class and then dividing the difference by the corresponding standard deviation, were averaged across years to calculate an index suitable for inclusion in models as an individual covariate, noted by AFS in models. Notation and selection of models followed common procedures in capture-recapture studies (e.g. [36]–[37]).

### Population modeling

#### Model building and assumptions

**Figure 6:**
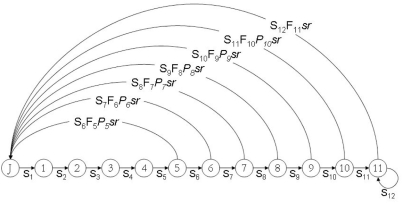
Life cycle of the bearded vulture study population with post-breeding census. The nodes show the different age-classes considered in the models: J for juveniles and S*_i_* for survival of each age-class *i*; *F*: fecundity (reproductive success); *sr*: sex ratio (0.5); P*_i_*: breeding probability of each age-class *i*. The model is only for females.

We built a predictive stochastic age-structured population model (only for females) to explore the functional dependence of λ (the population growth rate) on the demographic rates of the study population (see Fig. 6). This kind of perturbation analysis allows us to identify potential management targets because variations in demographic parameters with high sensitivity (or elasticity) produce large changes in λ. We also built a retrospective stochastic population model (also using the observed variability in vital rates, see [24]) to detect whether parameters used in the predictive model were reliable and described with certainty the behaviour of the population. Here, we used the same structure and number of replications as that described for the predictive model (see below), but run over the period for which data were collected (20 years), setting the initial population size to that estimated in 1985 (i.e. 37 breeding females).

#### Parameter estimations

Details on the effects of density-dependence on fecundity are well known [14] and can be introduced accurately in our model. Additional field data suggest that other parameters might also be affected by density-dependence in recent times, such as the proportion of breeders, which has decreased with time. Conversely, density-dependence was not considered in the recruitment curve, because of the scarce information available on this parameter (see below), or in survival rates because the relationship found was mainly attributed to the increase in poisoning instead of as a result of increments in population size (see Results). Here, we did not set any carrying capacity in the study system because 1) a large proportion of foraging resources are of human origin (AFS) and this can be modified in the future due to recent warnings on its usefulness [14], [39], and 2) it is difficult to assess how many breeding habitats are still available in the Pyrenees and neighbouring mountain ranges [16]. Our main interest from population modelling was thus to identify the most sensitive parameters of λ (particularly whether population projections predict high extinction probabilities), and not to predict how many pairs the study area can hold.

Although age-dependent recruitment proportion was unknown, field data showed that females bred for the first time between 6 and 12y of age [32]. Thus, our population model included 12 age-classes, assuming that full recruitment occurred at this age. Since recruitment in long-lived birds increases progressively with age and we know that up to 40% of sexually mature birds are not breeding [32], we distributed this proportion among our breeding age-classes (from 6 to 11y old) as follows: 20% in the first age-class (less likely breeders) and the remaining 20% equally among the other age-classes up to 11y (more likely breeders). This proportion of sexually mature, non-breeding birds was likely to be density-dependent, since saturation of habitat could increase the proportion of non-breeders (i.e., floater density, e.g. [40] to fit a density-dependent, non-linear function to the proportion of breeders, we assumed that (as recorded with fecundity, see below) it followed a negative exponential curve with a value of 0.4 for maximum density (at the end of the series) and 0 for minimum density (at the beginning of the series).

Estimates of fecundity (mean number of fledglings per pair = 0.52, SD = 0.5 with a binomial distribution because successful breeders raised a single chick) were obtained from 1,028 breeding events recorded in the study area during 1972–2002. Fecundity of bearded vultures was density-dependent, changing negatively not only with density of breeders but with total density [14]. From the field data, we fitted a negative exponential function (which fitted equally well as a logarithmic curve) rather than a linear function because: (a) it was more biologically plausible [41] and (b) the model fit better. Some data suggest that fecundity increases with experience in this species [34], but no information exists on fecundity variation with age. Thus, we did not correct fecundity by age in our age-structured model even though it likely increased with this individual covariate.

We introduced both environmental and demographic stochasticity in our model. The first takes into account temporal variability affecting demographic parameters annually, while the latter was introduced because of the small size (*N*) of the study population which could affect the binomial distribution, B(*N*, *p_i_*), of all vital rates *i* (see above). Since sample variance of survival estimates (i.e. *σ^2^*, a measure of error variation and covariance in *φ_i_*) was likely high due to the low number of individuals marked and resighted, we estimated only process variance (using MARK software [42]) to introduce it in simulations as a more reliable measure of environmental stochasticity.

#### Population trajectories

We ran Monte Carlo simulations using 500 replications of each combination of parameter values (see below, Table 2) to obtain potential population trajectories under different management scenarios (ULM software [43]). Extinction risk was projected on a time horizon of 50 years, which is commonly accepted as a long-term persistence period for bearded vultures following IUCN criteria for threatened species (ca. 3 generation times of the species [44]). Mean value of all trajectories together with its standard error was given for each year of the simulation. Thus, the probability of extinction at time *t*, *p_e_*(*t* = 50), was estimated as the ratio of the number of trajectories that have gone extinct after 50 years out of the total number of trajectories. The initial population size was set using the last census of the species in the Spanish Pyrenees (2007, 90 breeding females), and assuming that these females were equally distributed among the 7 breeding age-classes. We then added 60 non-breeding adult females distributed in the above-cited way among the same age-classes. Finally, 100 immature females were also added and distributed among the first 5 age-classes because 40% of the total population was estimated to be immature (authors unpubl. data).

### Management scenarios

One of the crucial environmental factors potentially severely affecting the viability of bearded vultures in the Pyrenees is the impact of poisoning, mainly through the ingestion of poisoned baits or medicated carcasses, when it affects adult survival rates (e.g. [18]). Even when the most important management action is to eradicate the illegal use of poison and change the quality of food supplied to most AFS, the employment of certain tools such as the opening of specific AFS (present results) to mitigate their effects was encouraged. Thus, we modelled potential scenarios combining different impacts of poisoning and AFS availability to disentangle their potential effects on population projections over time (see Table 2 for demographic parameters used).

#### Availability of artificial feeding sites

We first envisaged that poisoning continues affecting survival rates of vultures. Thus, we used the survival rates of pre-adult and adult birds estimated by our best selected model (scenario 1 in Table 2), which were lower than expected likely due to the effect of poisoning. The variance (once eliminated the variance from sampling error) around the mean value of the survival estimate was introduced in the model to reflect the environmental stochasticity of the poison impact, with no temporal autocorrelation (white environmental noise, e.g. [45]).

Most environmental fluctuations contain strong correlations at a multitude of scales [46]. In this case, the actual temporal dynamics of poisoning (mainly in the long-term) is unknown owing to the lack of field data available. However, strong eruptions of illegal poisoning associated with human-predator-prey conflicts are expected (author unpubl. data). Thus, we performed a second set of simulations (scenario 2, Table 2) considering that the temporal dynamics of poisoning are dominated by fluctuations, both short and long-term, with positive temporal autocorrelation (coloured environmental noise, e.g. [45]). Here, we used the actual temporal variability of survival probabilities φ (see Results) to estimate the coefficients (mean *a* and variance *b*) of the function φ_(t+1)_ = φ_(t)_·*a* +*rand*·*b* where *t* was the time (year), φ was the survival probabilities estimated in the previous CMR analysis and *rand* was a uniform distribution with range [0, 1]. The coefficient *a* has distribution ranges of [0<*a*<1] and [−1<*a*<0] for red (long-term) and blue (short-term) noise, respectively. Coefficients of this function were estimated by time series analysis using ARIMA (model AR(1)).

Finally, we simulated the behaviour of our population under effective management actions reducing the impact of poisoning (scenario 3 in Table 2), using actual adult survival estimates obtained in our study area but without poison effects (see Results).

#### No availability of AFS

This group of simulations allowed us to disentangle the actual usefulness of AFS to mitigate the effects of illegal poison on population persistence. First, we ran retrospective and prospective simulations of the population dynamics of bearded vultures in a scenario of poisoning and no availability of AFS (scenario 4 Table 2) to 1) assess the effectiveness of AFS given their observed trend during the last 20 years, and 2) evaluate the potential effectiveness of AFS on the expected trajectories of the study population. For these purposes, we estimated survival rates of young by fixing the beta parameter in the likelihood function estimated for the covariate “use of AFS” to 0 (see results). Survival estimates for adults was that estimated through our best CMR model (Table 1). Then, we simulated an alternative scenario in which there were no poison effects and AFS were not available. Here, survival estimates of adults were those obtained at the beginning of the study period (see Results) while survival of young was obtained as in the previous scenario of no AFS (scenario 5 Table 2).
